# Survey of general practitioners' knowledge about *Helicobacter pylori *infection

**DOI:** 10.1186/1471-230X-5-4

**Published:** 2005-01-26

**Authors:** Sevgi Canbaz, Ahmet Tevfik Sunter, Yildiz Peksen, Hakan Leblebicioglu

**Affiliations:** 1 Department of Public Health, Ondokuz Mayis University School of Medicine, Samsun, Turkey; 2 Department of Infectious Diseases and Clinical Microbiology, Ondokuz Mayis University School of Medicine, Samsun, Turkey

## Abstract

**Background:**

*Helicobacter pylori*, occurring throughout the world and causing gastroduodenal diseases, is one of the most common chronic bacterial agents in humans. The purpose of this study was to measure the general practitioners' (GPs) knowledge and practices pertaining to *H. pylori *infection.

**Methods:**

A cross-sectional type questionnaire survey was conducted in all of 19 primary health care centres (PHCC) in Samsun, Turkey, between November 1 and December 31, 2003. The questionnaire was sent to 124 GPs and 109 (87.9 %) of those filled in. They were requested to answer the questions on the knowledge, sources of medical information, diagnostic tests and treatment to *H. pylori*.

**Results:**

Medical journals were the most frequently used source of information on *H. pylori*, being cited by 86 (78.9%) of GPs. Ninety-two (84.4%) of the GPs reported having used one or more tests and 17 (15.6%) never used any test for the diagnosis of *H. pylori *infection. Only 9.8% had used stool antigen test for diagnosis. GPs reported that they would prescribe symptomatic treatment without ordering diagnostic tests for 29 (26.6%). 54.1% of the GPs explain that they sent patients with *H. pylori *infection to a specialist, and most used a triple drug regimen containing a PPI. Treatment duration varies between 7 to 28 days. 80.7 of the GPs treat patients for 14 days.

**Conclusion:**

GPs may not have enough knowledge about the importance of stool antigen test or possibility of usage of this test. GPs have not sufficient knowledge about the difference between symptomatic and asymptomatic individuals. It is thought that GPs preferred to treat the patients with suspected ulcer empirically or to send them to a specialist because of the limited diagnostic conditions. The efforts to educate the GPs about the algorithms regarding the management of *H. pylori *infection during post-graduation period should be improved in PHCCs.

## Background

*Helicobacter pylori*, occurring throughout the world and causing gastroduodenal diseases, is one of the most common chronic bacterial agents in humans [1]. Although the number of peptic ulcers unrelated to *H. pylori *is increasing, most ulcers are related to *H. pylori *infection [2,3]. For most of the patients with gastrointestinal symptoms apply to general practitioners (GPs), non-invasive "test and treat" policies for *H. pylori *infection have been promoted in order to improve early detection and treatment of ulcers in dyspeptic patients [4,5].

The successful isolation of *H. pylori *infection in patients with chronic gastritis and peptic ulcer disease in 1983 has fundamentally changed concept of the etiologic, pathogenesis and management of upper gastrointestinal (UGI) diseases [6]. This has led to an explosion of *H. pylori *related information and the development and publication of international, regional and national guidelines [7]. Subsequently, numerous educational initiatives have been undertaken to educate health care professionals regarding the appropriate diagnosis and management of this infection. However, results from several recent surveys conducted in different countries have revealed that significant confusion exists and discrepancies are present in the thinking among GPs with respect to the understanding of the relationship between *H. pylori *infection and the pathogenesis, diagnosis and treatment of UGI diseases [6]. The major uncertainties surround the management of patients with dyspepsia where the GPs needs to make a decision whether to test for *H. pylori *infection and treat if positive, and when to refer patients to a specialist [8,9].

It is thought that too many patients with dyspeptic symptoms apply to GPs in Turkey. This study was performed a survey of GPs' to assess their knowledge and practices pertaining to *H. pylori *infection.

## Methods

A cross-sectional study was conducted in all of 19 primary health care centres (PHCC) in Samsun, Turkey, between November 1 and December 31, 2003. The questionnaire was sent to all GPs (n = 124). 109 of 124 (87.9 %) GPs from different PHCCs completed the survey. The material used was adapted from the questionnaire devised by Sharma et al. [10] and translated into Turkish. The questionnaire was designed in a self-administered format with closed answers being provided to questions. Demographic variables such as gender, age and working year were assessed. The survey questionnaire includes a multiple-choice question relevant to sources of medical information. The *H. pylori *knowledge section contained 6 questions. The items assessed respondents' knowledge of diagnosis of infection, case selection for treatment and treatment options in *H. pylori*. Participating GPs were asked to offer the type(s) of diagnostic tests such as ELISA, histology, biopsy urease test (BUT), urea breath test (UBT) or culture of biopsy specimen.

A list of 7 different clinical presentations was given. It was asked whether the respondents would offer testing for *H. pylori *and also treat the infection when the test results were positive for *H. pylori*.

The respondents were asked to select a regimen for the management of *H. pylori *infection among the list of four drug combination regimens included proton pump inhibitor (PPI)-based triple therapies. [7,11,12]. GPs were also asked about their choice of treatment duration. The authors did not make any educational program focusing on *H. pylori *infection before or after the survey in this study period. Data were given as mean ± standard deviation (SD) and percentage.

## Results

### Sociodemographic characteristics

The mean age and working year of the GPs was 31.7 ± 5.4 and 7.2 ± 5.0 years, respectively; 59 (54.1%) of the GPs were women.

### Sources of information

Medical journals were the most frequently used source of information on *H. pylori*, being cited by 86 (78.9%) of GPs. Pharmaceutical company-sponsored symposia (70.6%), textbooks (64.2%), conferences (20.2%) and on-line sites (6.4%) were the other major source of information used by the GPs. These numbers add up to more than 100% because the GPs had been checked more than one item.

### Diagnostic tests for *H. pylori*

Ninety-two (84.4%) of the GPs reported having used one or more tests and 17 (15.6%) never used any test for the diagnosis of *H. pylori *infection. Of those, 44.1% had used UBT, 34.5% had used BUT, 23.8 % had used ELISA and 9.8% had used the stool antigen test. The practitioners included in the survey had not equally access to all diagnostic tests mentioned in the study.

### Testing and treatment choices for *H. pylori *infection

The proportions of GPs who would test patients for *H. pylori *infection in the 9 different clinical situations and the proportions of those who would offer treatment based on a positive test result, were summarized in Table-1. 92 (84.4%) of the GPs answered this section. GPs reported that they would prescribe symptomatic treatment without ordering diagnostic tests for 29 (26.6%). 54.1% of the GPs explain that they send patients with *H. pylori *infection to a specialist.

### Treatment of *H. pylori *infection in patients with confirmed *H. pylori*-positive

Treatment regimens of choice are listed in Table-2. Most used a triple drug regimen containing a PPI. Of the GPs 3.7% would treat patients for 7 days, 4.6% for 10 days, 80.7% for 14 days, 3.7% for 21 days, 5.5% for 28 days and 1.8% for more than 28 days.

## Discussion

UGI symptoms are common reasons for patients to visit GPs. In recent years, the development of non-invasive *H. pylori *detection methods, including ELISA and the UBT, has enabled GPs to diagnose and treat *H. pylori *infection. The inadequate treatment of peptic ulcer disease results in therapy failures, high recurrence rates, the emergence of resistant bacterial strains, and increased health care costs, therefore clinical application of current knowledge is crucial [13,14].

There are several tests used for diagnosis of *H. pylori *infection [12,13,15,16]. 84.4% the GPs surveyed used one or more tests for *H. pylori *infection same as Huang J et al.'s study [6]. However this was higher than reported in a recently preliminary survey of GPs, of whom 48% used one or more tests [17]. UBT is the most (44.1%) used tests in this study. On the other hand, stool antigen test, a useful test for diagnosis, was the least ordered test. It is thought that GPs may not have enough knowledge about the importance of stool antigen test or possibility of usage of this test. Diagnosis of infection should be done by using UBT or stool antigen test. It is always recommended to test by UBT, or endoscopy-based test if endoscopy is clinically indicated, for successful eradication. On the other hand stool antigen test is the alternative if UBT is not available [14].

It is worrisome that considerably fewer perceived a need for testing and subsequent treatment in patients with a new diagnosis and past history of duodenal ulcer (Table 1). Both the recent America College of Gastroenterology (ACG) publications [8] and Centers for Disease Control and Prevention (CDC) [7] clearly state that a new diagnosis and past history of duodenal ulcer disease is a definite indication for testing and, if positive, for treatment. Testing and treatment of *H. pylori *infection are recommended following resection of early gastric cancer and for low-grade gastric MALT lymphoma. Retesting after treatment may be prudent for patients with bleeding or otherwise complicated peptic ulcer disease [7].

Although it is not recommended to test the asymptomatic individuals for *H. pylori *infection, 64.1% of GPs reported that they would offer testing for *H. pylori *infection and 31.5% of them reported that they would treat *H. pylori *infection based on a positive test result in asymptomatic individuals. These findings suggest that GPs have not sufficient knowledge about the difference between symptomatic and asymptomatic individuals. On the other hand, in any person testing positive for the infection, treatment may be offered after a full discussion about its potential risks and benefits [4,18].

Anti-*H. pylori *therapy was almost never recommended for suspected ulcer disease without the prior use of diagnostic tests [13,18]. In this study it was found that 26.6% of GPs treat the initial onset of a suspected ulcer empirically without ordering diagnostic tests for *H. pylori *infection. 54.1% of the GPs, whether ordering diagnostic tests or not, explain that they send patients with suspected or diagnosed *H. pylori *infection to a specialist. In the light of these findings, it is thought that GPs preferred to treat the patients with suspected ulcer, empirically or to send them to a specialist because of the limited diagnostic conditions, the lack of rapidly diagnostic tests at PHCCs, or they thought that they should be treated by a specialist.

*H. pylori *peptic ulcers are treated with drugs that kill the bacteria, reduce stomach acid, and protect the stomach lining. Antibiotics are used to kill the bacteria. Two types of acid-suppressing drugs might be used: H_2 _blockers and PPI. H_2 _blockers and PPI have been prescribed alone for years as a treatment for ulcers. When used alone, these drugs do not eradicate *H. pylori *and, therefore, do not cure *H. pylori*-related ulcers. Bismuth subsalicylate, a component of Pepto-Bismol, is used to protect the stomach lining from acid. It also kills *H. pylori *[4,7,12,19].

The highest eradication rates are achieved with the following regimens: a PPI, clarithromycin, and either amoxicillin or metronidazole for 2 week; ranitidine bismuth citrate, clarithromycin, and either amoxicillin, metronidazole, or tetracycline for 2 week; a PPI, bismuth, metronidazole, and tetracycline for 1 to 2 week [7,11,12,20]. There is good evidence for the efficacy of 14-day triple regimens including a PPI or RBC. Possibility of shortening the duration of PPI-based triple therapies between 7 and 10 days will depend on further results from US-based studies. Seven-day duration may be too short. Some trials have failed to show a statistically significant difference in eradication rates between 7-day and 14-day duration [20]. 84.4% GPs test for *H. pylori *infection but 15.6% never test. Urea breath test is a commonly used investigative tool for *H. pylori *infection. Triple therapy consisting of a proton pump inhibitor, clarithromycin and amoxicillin is the most commonly used treatment combination for *H. pylori *infection.

It was found that most of the information was being obtained in traditional teaching formats such as medical journals, pharmaceutical company-sponsored symposia, textbooks and conferences. Other studies ([10,13,17]) confirmed our finding that medical journals were the most important source of information on *H. pylori *infection among GPs. Our data suggested that pharmaceutical company-sponsored symposia were used very frequently among GPs.

Patients with dyspeptic complaints are mostly managed in primary care. The most prescriptions for dyspepsia are empirical without testing due to limitations of diagnostic facilities around the world [21]. Similar results were reported that there were significant gaps related to testing and treating *H. pylori *infection [13,17].

## Conclusions

The diagnosis of and treatment of peptic ulcer disease related to *H. pylori *is not adequate. The choice of optimal therapeutic decision depends on the appropriate definition of the disease. In order to provide accurate diagnosis and treatment of *H. pylori *infection, it's suggested that efforts to educate the GPs about the algorithms regarding the management of *H. pylori *infection during post-graduation period should be improved in PHCCs.

## Competing interests

The author(s) declare that they have no competing interests.

## Authors' contributions

SC participated in the design and coordination of the study; ATS provided drafted the manuscript and performed the statistical analysis. YP drafted the questionnaire and participated in study design and coordination. HL conceived the study, participated in its design and drafted the manuscript. All authors read and approved the final manuscript.

## Pre-publication history

The pre-publication history for this paper can be accessed here:



## Figures and Tables

**Table 1:** The Proportions of GPs who would test patients for *H. pylori *infection in various clinical situations and those who would offer treatment based on a positive test (n = 92)

	**Would you offer testing for *H. pylori *infection**		**Would you treat *H. pylori *infection based on a positive test result**		**Current recommendation**	
	
Clinical presentation	**n**	**%**	**n**	**%**	**Test**	**Treat**
Peptic ulcer	73	*79.3*	74	*80.4*	yes	yes
New diagnosis of duodenal ulcer	67	*72.8*	73	*79.3*	yes	yes
Past history of duodenal ulcer	47	*51.1*	59	*64.1*	yes	yes
Asymptomatic; spouse has *H. pylori *infection	59	*64.1*	29	*31.5*	no	no
GERD*	38	*41.3*	50	*54.3*	no	no
Gastric cancer	30	*32.6*	19	*20.7*	no	no
Patient on long term PPI** treatment	43	*46.7*	29	*31.5*	no	no

*GERD = Gastroesophageal reflux disease

** PPI = proton pump inhibitor

**Table 2:** Treatment regimens

**Regimen**	Number*	**%**
PPI* + clarithromycin + amoxicillin	100	*91.7*
PPI + clarithromycin + metranidazole	7	*6.4*
Ranitidine bismuth citrate + clarithromycin + amoxicillin or metronidazole, or tetracycline	1	*0.9*
Ranitidine bismuth citrate + clarithromycin + amoxicillin or metronidazole, or tetracycline+PPI OR omeprazole	13	*11.9*

*The total number is higher than the number of the participants for the participants checked more than one item.

** PPI = proton pump inhibitor
